# Industrial robot application and total factor productivity of manufacturing enterprises

**DOI:** 10.1371/journal.pone.0331378

**Published:** 2025-09-24

**Authors:** Minghui Zhao, Zhenhua Wang, Shuangshuang Liu

**Affiliations:** 1 Beijing Friendship Hospital, Capital Medical University, Beijing, China; 2 Business School, University of International Business and Economics, Beijing, China; 3 Agricultural Information Institute, Chinese Academy of Agricultural Sciences, Beijing, China; 4 Center for International Agricultural Research, Chinese Academy of Agricultural Sciences, Beijing, China; Iowa State University, UNITED STATES OF AMERICA

## Abstract

In the context of intensifying global competitiveness and rapid technological advancement, industrial robots have emerged as a pivotal component in the integration of digital technology, exerting a vital influence on the transformation and enhancement of the manufacturing industry. The question of whether this transformative shift can significantly enhance total factor productivity (TFP) and accelerate the transformation of the manufacturing industry has attracted substantial academic attention. This study employs micro-panel data drawn from Chinese A-share listed manufacturing enterprises from 2007 to 2022 to examine the implications of industrial robot application on TFP and the underlying mechanisms. The results of our study indicate that industrial robots have a positive influence on TFP, and this effect persists over time. The results of the mechanism tests indicate that industrial robot application facilitates an increase in human capital, confirming their “talent aggregation effect”. Moreover, the application of industrial robots enhances enterprises’ innovative capabilities, thereby validating their “innovation effect”. Further examination of heterogeneity indicates that the enhancing impact of applying industrial robots on TFP is more pronounced among enterprises with high labor productivity, those that are state-owned, and enterprises operating in high-tech sectors. This research contributes to the understanding of the impact of industrial robot application on TFP, which is of considerable practical significance for emerging economies seeking to transform traditional enterprise factors, accelerate new technology integration, and steer the digital transformation of manufacturing.

## 1. Introduction

In the context of intensifying global competition and rapid technological advancements, the conventional large-scale, low-cost manufacturing model, which has been a fundamental aspect of traditional manufacturing, is facing challenges in adapting to the dynamic requirements of modern markets. The transformation of the manufacturing industry represents a central focus of current industrial development and is of utmost importance. It serves as a crucial catalyst for refining the global economic structure and represents a strategic pathway towards achieving sustainable development objectives and enhancing national competitiveness. As total factor productivity (TFP) serves as a vital metric for assessing the advancement of enterprises, enhancing TFP within manufacturing enterprises, and consequently promoting high-quality growth in the manufacturing industry has emerged as a critical issue for both industry practitioners and academic researchers. The accelerated pace of technological advancement and industrial transformation has created unprecedented opportunities for innovation within the manufacturing industry (Liu et al., 2020) [1]. Among these digital technologies, industrial robots are particularly noteworthy as innovative catalysts for industrial progress. For example, Tesla employs a highly automated production line that uses advanced robotic technology for body welding and assembly. These devices are able to complete complex welding tasks in a very short time. This not only improves production efficiency, but also significantly reduces labor costs and the incidence of human error, making the production capacity of its electric vehicles significantly increased. Therefore, conducting a comprehensive analysis of the impact of industrial robot application on the TFP of manufacturing enterprises, and the underlying mechanisms, holds considerable practical significance for boosting productivity, accelerating industrial transformation, and promoting high-quality development within the manufacturing industry.

A substantial body of research has been conducted to examine the economic impact of industrial robots. The principal insights derived from this research can be summarized as follows: Firstly, industrial robots, representing technological progress, have the capacity to displace low-skilled labor more significantly, generating a “substitution effect” (DeCanio, 2016; Acemoglu and Restrepo, 2020) [2,3]. This substitution effect will lead to a decrease in labor demand, resulting in widespread unemployment in the market (Autor et al., 2003; Frey and Osborne, 2017; Agrawal et al., 2019) [4–6]. Secondly, the application of industrial robots can lead to the creation of new employment opportunities, thereby stimulating enterprises to increase their demand for highly skilled personnel. This process optimizes the workforce structure and enhances enterprises’ innovation capacity, a phenomenon known as the “creation effect” (Graetz and Michaels, 2018; Balsmeier and Woerter, 2019; Koch et al., 2021) [7–9]. Lastly, the application of industrial robots exerts a diverse influence on the scale and composition of an enterprise’s workforce, demonstrating distinct effects across diverse types of enterprises. While the existing literature primarily concentrates on the macro-level labor market implications of industrial robots, there is insufficient examination of their impact on the total factor productivity of micro-enterprises. Duan et al. (2023) [10] examines how industrial robots affect enterprises’ TFP through these two aforementioned effects. However, further investigation is needed to determine whether additional mechanisms contribute to the consequences of applying industrial robots in micro-enterprises. This study aims to investigate the economic implications of industrial robots at the micro-enterprise level and provide compelling evidence for evaluating their function.

The contemporary theory of economic growth identifies the primary drivers for sustained economic growth as encompassing the level of technological progress, accumulation of human capital, and material production factors (Romer, 1990) [11]. High-quality development represents an economic model that efficiently provides society with superior products and services, characterizing it as a form of “intensive growth” achieved by enhancing the quality and efficiency of factor utilization (Brandt et al., 2012) [12]. The concept of TFP at the enterprise level measures the maximized output generated from various factor inputs such as capital, labor, technology, management, organizational structure, and other inputs. It serves as an indicator of efficiency in converting these factor inputs into final outputs (Hsieh and Klenow, 2009) [13].

Total factor productivity (TFP), which reflects the comprehensive productivity of all factors within an enterprise, is widely acknowledged by scholars as a pivotal indicator of high-quality growth. Prior research has provided a robust theoretical foundation and practical pathways for enhancing enterprises’ TFP. The existing literature has comprehensively analyzed the pathways to enhance TFP, with a primary focus on financial development (Sturm and Williams, 2004) [14], technological innovation (Baumann and Kritikos, 2016; Anzoategui et al., 2019) [15,16], and the labor market (Bennedsen et al., 2019) [17]. However, there is a significant deficiency in the existing literature regarding thorough analysis of the influence mechanisms exerted by artificial intelligence and automation technologies, particularly industrial robots, on enterprises’ TFP. As a paradigmatic manifestation of digital technological progress, industrial robots have the capacity to assist enterprises in reducing costs and increasing efficiency. Therefore, it is paramount to investigate the impact of industrial robot application on enterprises’ TFP. The objective of this study is to enhance our understanding of digital technology on enterprises’ TFP. Furthermore, it offers valuable insights for facilitating industrial transformation and fostering high-quality progression.

The Fourth Industrial Revolution has established digital technology as a crucial instrument for countries across the globe to drive the transformation and modernization of their manufacturing industry. China is currently at a critical juncture of economic transformation and upgrading. This presents both challenges and opportunities in terms of industrial structure adjustment and innovation-driven development. Currently, China is confronted with the challenge of a gradually diminishing demographic dividend, characterized by a decline in employment opportunities for low-skilled and repetitive labor. The resulting labor shortage has led to a notable increase in the cost of human capital within the manufacturing industry. Consequently, promoting intelligent and digital technologies has emerged as a novel trend in the context of manufacturing transformation. As the foremost manufacturing country globally, China boasts vast manufacturing industries that provide rich data resources for the application of industrial robots. In this context, examining the role of technological progress in catalyzing economic transformation and upgrading becomes significantly important.

This study utilizes panel data derived from Chinese A-share listed manufacturing enterprises, covering the period from 2007 to 2022, with the aim of achieving a comprehensive understanding of the implications of industrial robot application on enterprises’ TFP. The study examines both the relationship between industrial robot application and manufacturing enterprises’ TFP and the underlying mechanisms. The findings indicate that industrial robot application has a positive impact on enterprises’ TFP. This conclusion remains consistent even after conducting a series of robustness tests. Furthermore, an in-depth examination of the mechanisms suggests that industrial robot application can reshape enterprises’ production and operational structures, thereby attracting highly skilled personnel who are proficient in navigating this new technology. Consequently, there is an increase in human capital levels and its TFP, providing empirical support for the concept of a “talent aggregation effect”. Additionally, industrial robot application has been shown to enhance TFP by facilitating innovation capabilities, thus providing empirical support for the “innovation effect”. Moreover, heterogeneity analysis reveals considerable variation in the extent to which industrial robots enhance TFP across different enterprises. Notably, the impact of industrial robot application is more pronounced in high-labor productivity enterprises as well as state-owned and high-tech enterprises.

The findings of this study contribute to the existing body of research in the following ways:

Firstly, this research presents a comprehensive analysis of the impact of industrial robots on micro-enterprises, with a particular focus on total factor productivity. This endeavor builds upon and extends the existing academic literature on the economic implications of industrial robot application. Previous studies have predominantly focused on examining the “substitution effect” and “creation effect” of industrial robots on the macroeconomic labor market and employment rates. However, little attention has been paid to the impact of these robots on the decision-making processes of micro-enterprises, particularly in relation to their total factor productivity. The assessment of total factor productivity, defined as the efficiency with which an enterprise transforms inputs into outputs, is paramount for optimizing resource allocation within firms and fostering sustainable high-quality economic growth. Based on rigorous investigation, this study reveals that industrial robot application at the micro-enterprise level yields beneficial outcomes. This finding not only enhances research on the economic consequences of industrial robots but also provides compelling micro-level evidence for the effectiveness of digital technology in boosting enterprise productivity and fostering high-quality development. Furthermore, it offers invaluable insights for government policymakers in formulating strategies to accelerate enterprises’ transformation and modernization.

Secondly, the present study examines the impact of industrial robots on total factor productivity in enterprises within the context of digital technology. This contributes to and develops the existing analysis of factors influencing enterprise TFP. Prior studies have predominantly focused on identifying determinants of enterprises’ TFP, including financial growth (Sturm and Williams, 2004) [14], technological innovation (Baumann and Kritikos, 2016; Anzoategui et al. 2019) [15,16], and labor market (Bennedsen et al. 2019) [17]. However, the complex mechanisms through which industrial robots affect enterprises’ TFP have been largely overlooked in existing literature. The findings suggest that industrial robots have the potential to enhance the structure of enterprises’ human capital, thereby creating a positive aggregation effect regarding talent. Moreover, as a significant catalyst for technological advancement, industrial robots serve as a crucial driver of enterprises’ innovation capabilities. This research contributes to understanding enterprises’ TFP from a digital technology perspective and provides actionable insights for manufacturing industry to develop strategies for substituting human labor with machine-based processes and enhancing their competitive advantage in the market.

Thirdly, this study conducts a comprehensive examination of the mechanisms through which industrial robots affect TFP. The findings of our research indicate that the application of industrial robots has a positive impact on enterprises’ TFP primarily by promoting talent aggregation and enhancing innovation capabilities. Although some researchers have examined the influence of industrial robot application on TFP (Duan et al., 2023; Xu et al., 2024; Qin et al., 2025) [10,18,19], there is a notable absence of studies investigating these impacts from the perspective of technical and labor production factors. The objective of this research is to address this knowledge gap by investigating the fundamental mechanisms through which industrial robot application affects TFP, thereby making a significant contribution to the existing academic discourse. The aim is to clarify the complex relationship between industrial robots and TFP, thus supporting both the “talent aggregation effect” and “innovation effect” associated with industrial robots.

## 2. Hypothesis development

The improvement of TFP is crucial for enterprises seeking sustainable development and market competitiveness, as it drives economic growth and facilitates sustained expansion (Solow, 1956) [20]. Enhancing TFP is not only essential for microeconomic entities to refine resource allocation and improve operational efficiency but also a catalyst for achieving high-quality growth and transformative upgrading at the macroeconomic level. TFP represents a comprehensive metric that evaluates an enterprise’s maximum output with a given set of inputs, leading to improvements in both production efficiency and economic returns. Increasing the total factor productivity of enterprises effectively promotes industrial structure optimization, economic transformation, and upgrading. In the context of rapid globalization and the growing role of information technology, enhancing the total factor productivity of enterprises, as fundamental units within national economies, holds great significance in improving national competitiveness and achieving sustainable economic development.

The relentless advancement of big data, artificial intelligence, the Internet of Things, and associated technologies has resulted in a profound integration between digital technology and the real economy. Consequently, industrial robots are increasingly replacing repetitive traditional manual work and assuming more responsibilities, thereby marking a transformation that is set to enhance enterprises’ TFP. Firstly, the application of industrial robots has prompted a transformation in the workforce structure, shifting from an operational and skill-centric model to a knowledge-centric one (Du and Lin, 2022) [21]. This transition inevitably leads to an increase in the skill requirements for the workforce, resulting not only in elevated demand for highly skilled personnel but also in a greater proportion of such skilled workers within enterprises. Subsequently, this optimizes the composition of the workforce and enhances the quality of human capital. Secondly, the integration of cutting-edge technologies into industrial robotics stimulates innovation in technology research and development, production processes, and product-related services. This integration results in more efficient allocation of resources and enhanced production efficiency (Makridakis, 2017; Graetz and Michaels, 2018) [22,23]. Moreover, their intelligent functionalities facilitate the modernization of enterprises’ organizational frameworks and management methodologies, thereby creating an environment conducive to sustained innovation. The aggregation of talent associated with industrial robots increases the density of knowledge networks and facilitates knowledge transfer, fostering the growth of intra-group innovation networks. Ultimately, this drives continuous innovation and transformation leading to a sustained enhancement of total factor productivity (Che and Zhang, 2018) [24]. Based on the analysis above, this paper formulates the following research hypothesis:

**Hypothesis 1:** The industrial robot application can enhance the total factor productivity of enterprises.

A theoretical examination has been conducted to determine the direct implications of industrial robot application on enterprises’ total factor productivity. The following analysis will examine in greater detail the mechanisms underlying this influence, with a particular focus on two key areas: talent aggregation and innovation capacity.

The implementation of industrial robots has had a significant impact on the production and operational structure of enterprises, as evidenced by substantial restructuring. This transformation has facilitated the recruitment of highly skilled employees who are aligned with the changes brought about by industrial robot application. Consequently, enterprises have been able to enhance their human capital, resulting in an increase in their TFP.

Firstly, the automation and intelligence inherent in industrial robots have facilitated the refinement of internal talent structures within enterprises, enabling the convergence of highly skilled individuals in pivotal roles and creating a talent aggregation effect. The application of industrial robots has accelerated technological advancements, optimized resource allocation, and alleviated the burden on employees engaged in arduous repetitive tasks (Gaggl and Wright, 2017; Graetz and Micheals, 2018) [23,25]. Consequently, these employees can now devote more time to tasks that require advanced cognitive and creative abilities (Goos et al., 2009; Michaels et al., 2014) [26,27]. Exceptionally skilled individuals are crucial for innovation as they possess exceptional professional skills, robust innovative capabilities, and adept knowledge application abilities. Exceptionally skilled individuals are capable of quickly comprehending and effectively utilizing advanced technologies, thereby amplifying the spillover effects of technology within enterprises (Jung and Lim, 2020) [28]. The rise of such individuals reinforces the labor force structure, elevates human capital levels, and injects new vitality into innovation and development. As a result, this leads to increased innovation and promotes technological progress (Aghion et al., 2015; Wang et al., 2020) [29,30]. Furthermore, the application of industrial robots can facilitate the creation of employment opportunities with distinctive advantages, thereby generating new forms of attractive employment and work modalities for highly educated professionals. For example, the use of industrial robots may lead to an increased demand for innovative roles such as robot engineers. This prompts enterprises to enhance their recruitment and training activities in relevant domains, thereby improving the caliber and competitiveness of their human resources. The appealing remuneration packages and career progression opportunities brought by industrial robots can attract and retain a larger number of exceptional talents, thus establishing a virtuous cycle. Based on the above analysis, this paper formulates the following research hypothesis:

**Hypothesis 2:** The industrial robot application enhances total factor productivity by facilitating the aggregation of talent.

Technological advancement is the key to enhancing the total factor productivity of enterprises, as it helps refine the distribution of production elements and upgrade the technological proficiency of enterprises, thereby having a favorable impact on TFP enhancement. Among the key factors influencing the growth of innovation capacity, industrial robot application plays a significant role and has a considerable impact on the enhancement of TFP.

Firstly, the application of industrial robots serves to stimulate innovation within the sphere of production technologies. Enterprises persistently focus on researching, developing, and deploying industrial robots in order to explore new technologies, processes, and methodologies. This not only enhances the accuracy and efficiency of product manufacturing but also fosters the infiltration and integration of new technologies across various stages including product design, production workflows, and quality assurance. As a result, technological innovation becomes more dynamic. The synergistic application of cutting-edge manufacturing technologies with industrial robots has been shown to enhance production precision, reduce energy consumption, and optimize production workflows thereby propelling continual advancements in production technologies (Loebbecke and Picot, 2015; Acemoglu and Restrepo, 2020) [3,31]. This technological innovation serves to enhance enterprises’ production efficiency and afford them heightened competitive advantages in the marketplace thereby contributing to the enhancement of TFP (Balsmeier and Woerter, 2019) [8]. Secondly, the application of industrial robots serves to stimulate innovation in enterprises’ management practices. In order to optimize the efficacy of industrial robots, it is essential for enterprises to continuously refine their production management processes, increase the level of digitization, and strengthen interdepartmental collaboration. By enhancing knowledge accumulation and integrating both internal and external knowledge resources, enterprises ultimately achieve the goals of optimizing production workflows, improving product quality, and increasing innovation capabilities (Huang et al., 2017) [32]. Concurrently, the application of industrial robots facilitates a reduction in resource and energy consumption as well as production costs (Impullitti et al., 2022) [33], an increase in output per unit of input, and consequently enhances TFP. Based on the above analysis, this paper formulates the following research hypothesis:

**Hypothesis 3:** The industrial robot application enhances total factor productivity by improving their innovative capabilities.

## 3. Methodology and data

### 3.1. Sample and data sources

The current research conducted an examination on manufacturing enterprises listed on China’s A-share market, covering the period from 2007 to 2022. The data related to industrial robots were obtained from the International Federation of Robotics (IFR), which conducts annual surveys of global robot manufacturers, resulting in a comprehensive “country-industry-year” database. By using the first two industry codes in conjunction with those provided by the IFR, we matched the data of listed companies with the industrial robot data. Financial and productivity details for these listed companies were extracted from the China Stock Market & Accounting Research database. Our sample excluded non-manufacturing companies, as well as Special Treatment and Particular Transfer listed companies, those with negative owner’s equity, and entities with missing data observation values. To reduce the influence of outlier values, all continuous variables were winsorized at the 1st and 99th percentile levels. Ultimately, our final sample comprised 16,103 observations.

### 3.2. Variable definition

#### 3.2.1. Dependent variable.

The dependent variable under investigation is the Total Factor Productivity (TFP) of enterprises. This metric not only embodies the efficiency of their input-output operations but also serves as an indication of their innovation-oriented capabilities and growth potential (Brandt et al., 2012) [12]. Following the methodologies of Levinsohn and Petrin (2003) [34], this paper employs two methods to measure the TFP of enterprises: TFP calculated using the Levinsohn-Petrin method (TFP_LP) and TFP calculated using the fixed effects method (TFP_FE). The TFP_LP approach controls for potential endogeneity between input choices and unobserved productivity by using intermediate input demand (e.g., material costs) as a proxy. And, the TFP_FE approach estimates productivity as the residual from a Cobb-Douglas production function with firm-level fixed effects, which absorbs time-invariant unobservable heterogeneity across firms.

#### 3.2.2. Independent variable.

The independent variable is the application of industrial robots (ROBOT). Following the research of Acemoglu and Restrepo (2020) [3], the level of industrial robot application is measured by the penetration rate of industrial robots at the enterprise level. The specific calculation process is as follows:

First, the industry-level industrial robot penetration was calculated using formula (1):


$${\text{PR}}_{\text{jt}}=\frac{{\text{IR}}_{\text{jt}}}{{\text{L}}_{\text{j},\text{t}=\text{2008}}}$$ (1)

Where ${\text{IR}}_{\text{jt}}\;$signifies the installation of industrial robots in Chinese industrial sector j during year t. ${\text{L}}_{\text{j},\text{t}=\text{2008}}$ represents the number of workers in Chinese industry j during the base period of 2007. The year 2008 was the base year t. ${\text{PR}}_{\text{jt}}$ denotes the penetration of industrial robots within the Chinese industrial sector j in year t.

Second, firm-level industrial robotics penetration is calculated using Equation (2):


$${\text{ROBOT}}_{\text{ijt}}=\frac{{\text{AE}}_{\text{ijt}}}{{\text{MAE}}_{\text{t}}}*{\text{PR}}_{\text{jt}}$$ (2)

Where ${\text{ROBOT}}_{\text{ijt}}$ denotes the industrial robot penetration of firm i in industrial sector j within the manufacturing industry in year t. ${\text{AE}}_{\text{ijt }}$ denotes asset expenditures, such as fixed and intangible assets, of firm i in industry sector j within the manufacturing industry in year t. ${\text{MAE}}_{\text{t}}\;$ denotes the median asset expenditures of all firms in the manufacturing industry in year t.

#### 3.2.3. Control variables.

Drawing on previous research (Acemoglu and Restrepo, 2020; Liang et al., 2023) [3,35], this paper has selected a set of variables that reflect corporate financial and governance conditions as control variables. These include company size (SIZE), return on assets (ROA), Tobin’s Q (TOBINQ), firm age (AGE), cash flow ratio (CASH), gross profit margin (GPM), management expense ratio (MFEE), tangible asset ratio (TANG), as well as industry and year variables. The definitions of each variable are presented in Table 1.

**Table 1:** Variable definitions.

Variables	Definition
TFP_LP	TFP calculated using the Levinsohn-Petrin method
TFP_FE	TFP calculated using the fixed effects method
ROBOT	The penetration of firms industrial robots.
SIZE	The natural logarithm of total market value.
ROA	The ratio of net profit to year-end total assets
TOBINQ	The ratio of a company’s market value to its total assets
AGE	The age of the company since its establishment
CASH	The ratio of net cash flow from operating activities to total assets
GPM	The ratio of gross profit to operating revenue
MFEE	The ratio of administrative expenses to operating revenue
TANG	The ratio of net fixed assets to total assets
Industry	Industry dummy Variables
Year	Year dummy Variables

### 3.3. Model specification

#### 3.3.1. Baseline model.

In order to verify the effect of industrial robots on the total factor productivity of enterprises, refers to the research of Liang et al.(2023) [35], we construct the following baseline regression model (3):


$${TFP}_{i,t}=\beta_{\text{0}}+\beta_{\text{1}}{ROBOT}_{i,t}+\sum {CONTROLS}_{i,t}+\varepsilon_{i,t}\;$$ (3)

where ${TFP}_{i,t}$ is the dependent variable, denoting total factor productivity of enterprises. ${ROBOT}_{i,t}$ is the independent variable, denoting industrial robot application. ${CONTROLS}_{i,t}$ represents all control variables and industry-year fixed effects. $\varepsilon_{i,t}$ represents the random disturbance term. All regression analyses conducted in this paper are clustered at the firm level to mitigate potential issues of heteroskedasticity and autocorrelation. The primary focus is on examining the regression coefficient associated with the independent variable, industrial robot application. If this coefficient is found to be significantly greater than 0, it suggests that the impact of industrial robot application on enterprises’ total factor productivity is significantly positive. This implies that an increase in the extent of industrial robot application enhances the total factor productivity of enterprises, thereby confirming Hypothesis 1.

## 4. Empirical analysis and results

### 4.1. Descriptive statistics and correlation analysis

The descriptive statistics for the primary variables under consideration are presented in Table 2. It is evident from the table that there is a significant level of industrial robot penetration within enterprises, with an average (median) prevalence of 1.8491 (1.5393). Therefore, it is important to examine the impact of industrial robot penetration on total factor productivity (TFP) in enterprises. The mean (median) TFP, calculated using the LP method, is 8.9277 (8.8361), while the corresponding values obtained using the fixed-effects method are 11.3640 (11.2375). Additionally, both methods indicate variability in productivity across different enterprises with regards to TFP – 0.9969 for LP and 1.2298 for fixed-effects method.

**Table 2:** Descriptive statistics.

VARIABLES	N	MEAN	SD	MIN	P25	P50	P75	MAX
ROBOT	16103	1.8491	1.5137	0.0000	0.6286	1.5393	2.7119	6.5820
TFP_LP	16103	8.9277	0.9969	6.8729	8.2409	8.8361	9.5052	11.7268
TFP_FE	16103	11.3640	1.2298	8.8742	10.5101	11.2375	12.0647	14.8751
SIZE	16103	22.7040	0.9847	20.8850	22.0014	22.5810	23.2476	25.7332
ROA	16103	0.0386	0.0602	−0.2333	0.0133	0.0371	0.0680	0.1956
TOBINQ	16103	2.0854	1.2430	0.9054	1.2966	1.6827	2.3936	8.0118
AGE	16103	16.9860	5.3364	5.0000	13.0000	17.0000	20.0000	31.0000
CASH	16103	0.1769	0.1199	0.0190	0.0918	0.1450	0.2288	0.5883
GPM	16103	0.0536	0.1397	−0.6434	0.0103	0.0549	0.1188	0.3960
MFEE	16103	0.0900	0.0599	0.0117	0.0499	0.0772	0.1128	0.3593
TANG	16103	0.2380	0.1383	0.0191	0.1322	0.2131	0.3203	0.6306

Note: The table provided here presents the descriptive statistics related to the application of industrial robots, total factor productivity, and the control variable discussed in this paper. The sample includes 16,103 observations spanning from 2007 to 2022.

Table 3 presents the correlation coefficients between variables. Spearman correlation coefficients are located above the principal diagonal in the correlation matrix, while Pearson correlation coefficients are situated below it. According to the Pearson correlation coefficients, there is a significant positive correlation between industrial robot penetration (ROBOT) and total factor productivity (TFP). This finding suggests that applying industrial robots enhances enterprises’ total factor productivity to some extent, providing initial support for Hypothesis H1 proposed in this research. The interpretation of Spearman’s correlation coefficients aligns with the above and will not be reiterated here.

**Table 3:** Correlation coefficient.

	ROBOT	TFP_LP	TFP_FE	SIZE	ROA	TOBINQ	AGE	CASH	GPM	MFEE	TANG
ROBOT	1	0.4413***	0.4922***	0.5302***	−0.0490***	−0.2023***	0.2876***	−0.1850***	−0.0497***	−0.1307***	0.1257***
TFP_LP	0.4816***	1	0.9828***	0.7028***	0.1066***	−0.3522***	0.1696***	−0.0733***	−0.0426***	−0.5850***	−0.0230***
TFP_FE	0.5302***	0.9852***	1	0.7396***	0.0633***	−0.3881***	0.1740***	−0.1301***	−0.0677***	−0.5634***	0.1039***
SIZE	0.5485***	0.7501***	0.7870***	1	0.1319***	0.0062	0.1714***	−0.0761***	0.1042***	−0.1790***	−0.0288***
ROA	−0.0456***	0.1367***	0.1019***	0.1671***	1	0.2708***	−0.0601***	0.2774***	0.8420***	−0.0557***	−0.1721***
TOBINQ	−0.1901***	−0.2902***	−0.3192***	0.0531***	0.1719***	1	−0.0082	0.1356***	0.2326***	0.3404***	−0.1344***
AGE	0.2665***	0.1573***	0.1615***	0.1660***	−0.0568***	0.0323***	1	−0.0843***	−0.0966***	−0.0928***	−0.0063
CASH	−0.1936***	−0.1052***	−0.1584***	−0.0757***	0.2583***	0.1238***	−0.1003***	1	0.2826***	0.0848***	−0.3560***
GPM	−0.0490***	0.0819***	0.0551***	0.1214***	0.7874***	0.0873***	−0.0847***	0.2743***	1	0.0511***	−0.1802***
MFEE	−0.1443***	−0.5513***	−0.5339***	−0.1746***	−0.1786***	0.3158***	−0.0606***	0.1063***	−0.2515***	1	−0.1519***
TANG	0.1181***	0.0012	0.1244***	−0.0001	−0.1575***	−0.1152***	−0.0063	−0.3657***	−0.1401***	−0.1674***	1

Note: This table presents the correlation coefficient among the main variables in This paper. *, **, *** indicate statistical significance at the 10%, 5%, and 1% levels, respectively, using a two-tailed t-test.

### 4.2. Baseline regressions

Table 4 presents the results of the baseline regression analysis, which investigates the correlation between industrial robot application and TFP. Specifically, columns (1) to (3) showcase the estimated TFP results obtained using the Levinsohn-Petrin (LP) method, while columns (4) to (6) display the estimated TFP outcomes derived from the fixed effects approach. In particular, columns (1) and (4) present regression results without any control variables and without considering fixed effects for industry and year. Columns (2) and (5) reveal regression results that exclude control variables but incorporate fixed effects for industry and year. Columns (3) and (6) present regression results that include control variables and account for both industry and year fixed effects. Across all columns from (1) to (6), consistently demonstrate that the regression coefficients for industrial robots are significantly positive at a 1% level, suggesting that their application notably enhances firm-level TFP thus supporting Research Hypothesis 1. Furthermore, computed VIF values for all models are below 10 indicating no issues with multicollinearity.

**Table 4:** Baseline regressions.

	(1)	(2)	(3)	(4)	(5)	(6)
VARIABLES	TFP_LP	TFP_LP	TFP_LP	TFP_FE	TFP_FE	TFP_FE
ROBOT	0.3172***	0.5957***	0.0660***	0.4308***	0.8420***	0.1285***
	(0.0116)	(0.0138)	(0.0103)	(0.0139)	(0.0146)	(0.0111)
SIZE			0.6656***			0.8504***
			(0.0123)			(0.0130)
ROA			1.9144***			1.9291***
			(0.2252)			(0.2388)
TOBINQ			−0.1395***			−0.1938***
			(0.0071)			(0.0077)
AGE			0.0077***			0.0089***
			(0.0017)			(0.0017)
CASH			−0.1121*			−0.1704***
			(0.0613)			(0.0646)
GPM			−1.2611***			−1.3477***
			(0.1164)			(0.1241)
MFEE			−6.4029***			−6.6638***
			(0.2043)			(0.2139)
TANG			−1.0287***			−0.2291***
			(0.0713)			(0.0743)
Constant	8.3413***	8.7248***	−4.9512***	10.5674***	11.1012***	−6.7576***
	(0.0252)	(0.0737)	(0.2825)	(0.0301)	(0.0809)	(0.2984)
Observations	16,103	16,103	16,103	16,103	16,103	16,103
R-squared	0.2319	0.4610	0.8255	0.2811	0.5780	0.8708
Industry	NO	YES	YES	NO	YES	YES
Year	NO	YES	YES	NO	YES	YES

Note: This table presents the regression results of the impact of industrial robot adoption on the total factor productivity. *, **, *** indicate significance at the 10%, 5%, and 1% levels, respectively. Standard errors adjusted by firm clustering are in brackets. The same applies to subsequent tables.

### 4.3. Robustness test

To further enhance the robustness of the findings, this section conducts a series of robustness tests, including replacing both the independent and dependent variables, changing the regression model, conducting persistence tests, eliminating the influence of other factors, and performing IV estimation.

#### 4.3.1. Change variables.

Firstly, we conduct a validation test by substituting the dependent variable. Specifically, we choose to use Total Factor Productivity estimated through the Ordinary Least Squares method, referred to as TFP_OLS, instead of the TFP values originally calculated using the LP and fixed-effects methods in our baseline regression model. The results of this verification exercise are presented in column (1) of Table 5, indicating a significant positive relationship between the regression coefficient and industrial robot application. Secondly, we perform a second test involving the substitution of the independent variable. In this case, we use the count of operational industrial robots (ROBOT2) as an alternative measure to replace the penetration rate of industrial robots used in the baseline analysis. The outcomes of this test are shown in columns (2) and (3) of Table 5. Examination of these columns reveals that both estimated coefficients associated with industrial robot application are significantly positive.The above empirical findings confirm that using substitute variables yields results that align substantially with those obtained from the baseline regression, thereby reinforcing the robustness of conclusions presented in this study. Furthermore, we replace the original variables with the number of changes in the robots (ROBOT3) and the research results still support our conclusion in columns (4) and (5) of Table 5.

**Table 5:** Change variables.

VARIABLES	(1)	(2)	(3)	(4)	(5)
	TFP_OLS	TFP_LP	TFP_FE	TFP_LP	TFP_FE
ROBOT	0.1153***				
	(0.0108)				
ROBOT2		0.0606***	0.1271***		
		(0.0101)	(0.0109)		
ROBOT3				0.0257**	0.1119***
				(0.0112)	(0.0128)
SIZE	0.8080***	0.6628***	0.8369***	0.0055*	0.0065**
	(0.0127)	(0.0129)	(0.0137)	(0.0028)	(0.0031)
ROA	1.9008***	1.9116***	1.9318***	0.5727***	0.6406***
	(0.2324)	(0.2247)	(0.2378)	(0.0819)	(0.0869)
TOBINQ	−0.1816***	−0.1385***	−0.1895***	0.0050**	0.0017
	(0.0075)	(0.0071)	(0.0078)	(0.0024)	(0.0024)
AGE	0.0085***	0.0076***	0.0088***	−0.0019***	−0.0029***
	(0.0017)	(0.0017)	(0.0017)	(0.0005)	(0.0005)
CASH	−0.1564**	−0.1029*	−0.1498**	−0.1666***	−0.1557***
	(0.0631)	(0.0614)	(0.0647)	(0.0252)	(0.0256)
GPM	−1.3210***	−1.2605***	−1.3425***	0.2358***	0.2910***
	(0.1209)	(0.1162)	(0.1234)	(0.0393)	(0.0420)
MFEE	−6.5877***	−6.3992***	−6.6492***	−0.5798***	−0.5189***
	(0.2097)	(0.2043)	(0.2135)	(0.0615)	(0.0620)
TANG	−0.3590***	−1.0481***	−0.2966***	−0.2091***	−0.1585***
	(0.0727)	(0.0742)	(0.0777)	(0.0227)	(0.0232)
Constant	−6.3317***	−4.9027***	−6.4847***	−0.0273	−0.0250
	(0.2915)	(0.2936)	(0.3114)	(0.0678)	(0.0735)
Observations	16,103	16,103	16,103	13,256	13,256
R-squared	0.8649	0.8254	0.8711	0.1532	0.1861
Industry	YES	YES	YES	YES	YES
Year	YES	YES	YES	YES	YES

#### 4.3.2. Change models.

To ensure the reliability of our research findings, we conducted additional analyses using various specifications of the fixed-effects model. The results of these analyses are presented in columns (1) to (4) of Table 6. Specifically, columns (1) and (2) display the results that incorporate fixed effects for both companies and years, while columns (3) and (4) show the outcomes with fixed effects for industries, years, and provinces. The findings reveal that the coefficient estimates associated with industrial robot application exhibit significant positivity, consistent with the baseline regression results. This consistency underscores the robustness of our research conclusions.

**Table 6:** Change models.

VARIABLES	(1)	(2)	(3)	(4)
	TFP_LP	TFP_FE	TFP_LP	TFP_FE
ROBOT	0.0337***	0.0764***	0.0685***	0.1304***
	(0.0086)	(0.0093)	(0.0101)	(0.0109)
SIZE	0.4614***	0.6152***	0.6638***	0.8499***
	(0.0152)	(0.0166)	(0.0121)	(0.0128)
ROA	0.5185***	0.4397***	1.8624***	1.8739***
	(0.1263)	(0.1272)	(0.2157)	(0.2285)
TOBINQ	−0.0830***	−0.1239***	−0.1387***	−0.1928***
	(0.0050)	(0.0056)	(0.0068)	(0.0075)
AGE	−0.0180	−0.0155	0.0087***	0.0099***
	(0.0161)	(0.0180)	(0.0016)	(0.0017)
CASH	−0.3218***	−0.4050***	−0.1263**	−0.1821***
	(0.0391)	(0.0420)	(0.0592)	(0.0624)
GPM	−0.2257***	−0.2556***	−1.2713***	−1.3578***
	(0.0648)	(0.0657)	(0.1107)	(0.1182)
MFEE	−4.5999***	−4.7866***	−6.3538***	−6.6086***
	(0.1690)	(0.1732)	(0.1961)	(0.2056)
TANG	−0.8794***	−0.1030	−1.0086***	−0.2130***
	(0.0649)	(0.0654)	(0.0707)	(0.0738)
Constant	−0.5470	−1.7100***	−4.8044***	−6.6481***
	(0.3842)	(0.4212)	(0.2787)	(0.2943)
Observations	16,103	16,103	16,103	16,103
R-squared	0.7089	0.7662	0.8305	0.8740
Firm	YES	YES	NO	NO
Year	YES	YES	YES	YES
Industry	NO	NO	YES	YES
Province	NO	NO	YES	YES

#### 4.3.3. Cluster at the city level.

The standard errors are currently clustered at the firm level. We also try clustering at the city level to account for autocorrelation in error terms among firms in the same city. The results of these analyses are presented in columns (1) to (6) of Table 7. The findings reveal that the coefficient estimates associated with industrial robot application exhibit significant positivity, consistent with the baseline regression results.

**Table 7:** Cluster at the city level.

VARIABLES	(1)	(2)	(3)	(4)	(5)	(6)
	TFP_LP	TFP_LP	TFP_LP	TFP_FE	TFP_FE	TFP_FE
ROBOT	0.3172***	0.5957***	0.0660***	0.4308***	0.8420***	0.1285***
	(0.0137)	(0.0148)	(0.0105)	(0.0174)	(0.0152)	(0.0112)
SIZE			0.6656***			0.8504***
			(0.0143)			(0.0146)
ROA			1.9144***			1.9291***
			(0.2218)			(0.2369)
TOBINQ			−0.1395***			−0.1938***
			(0.0080)			(0.0084)
AGE			0.0077***			0.0089***
			(0.0014)			(0.0015)
CASH			−0.1121*			−0.1704***
			(0.0632)			(0.0646)
GPM			−1.2611***			−1.3477***
			(0.1211)			(0.1274)
MFEE			−6.4029***			−6.6638***
			(0.2647)			(0.2695)
TANG			−1.0287***			−0.2291**
			(0.0984)			(0.0967)
Constant	8.3413***	8.7248***	−4.9512***	10.5674***	11.1012***	−6.7576***
	(0.0281)	(0.0847)	(0.3313)	(0.0371)	(0.0923)	(0.3410)
Observations	16,103	16,103	16,103	16,103	16,103	16,103
R-squared	0.2319	0.4610	0.8255	0.2811	0.5780	0.8708
Industry	NO	YES	YES	NO	YES	YES
Year	NO	YES	YES	NO	YES	YES

#### 4.3.4. Persistent test.

We investigate the long-term influence of industrial robots on total factor productivity by lagging the dependent variable by one and two time periods. The resulting estimation results are displayed in Table 8. Specifically, columns (1) and (2) present the estimates for the dependent variable one period ahead, while columns (3) and (4) illustrate the findings two periods ahead. The outcomes reveal that all coefficient estimates associated with industrial robot application are significantly positive. This provides additional evidence that the beneficial impact of industrial robot application on enterprises’ TFP persists over time, supporting the conclusion derived from the baseline regression analysis.

**Table 8:** Persistent test.

VARIABLES	(1)	(2)	(3)	(4)
	TFP_LP_t + 1_	TFP_FE_t + 1_	TFP_LP_t + 2_	TFP_FE_t + 2_
ROBOT	0.0553***	0.1083***	0.0541***	0.1005***
	(0.0111)	(0.0119)	(0.0131)	(0.0142)
SIZE	0.6792***	0.8723***	0.6753***	0.8720***
	(0.0130)	(0.0136)	(0.0147)	(0.0154)
ROA	2.8162***	2.8415***	3.0522***	3.1041***
	(0.3066)	(0.3279)	(0.4060)	(0.4401)
TOBINQ	−0.1330***	−0.1882***	−0.1243***	−0.1799***
	(0.0080)	(0.0086)	(0.0094)	(0.0102)
AGE	0.0051***	0.0055***	0.0025	0.0021
	(0.0019)	(0.0019)	(0.0022)	(0.0022)
CASH	−0.1641**	−0.2444***	−0.0780	−0.1481*
	(0.0686)	(0.0717)	(0.0780)	(0.0820)
GPM	−1.5577***	−1.5990***	−1.8312***	−1.8681***
	(0.1508)	(0.1607)	(0.2026)	(0.2189)
MFEE	−5.8795***	−6.1584***	−5.2706***	−5.6062***
	(0.2224)	(0.2308)	(0.2512)	(0.2638)
TANG	−0.8202***	−0.1197	−0.6178***	0.0067
	(0.0767)	(0.0800)	(0.0878)	(0.0925)
Constant	−5.3638***	−7.2825***	−5.1817***	−7.1170***
	(0.2977)	(0.3108)	(0.3356)	(0.3499)
Observations	13,256	13,256	10,958	10,958
R-squared	0.7878	0.8436	0.7389	0.8026
Industry	YES	YES	YES	YES
Year	YES	YES	YES	YES

#### 4.3.5. Eliminate the influence of other factors.

To ensure the rigor and scientific validity of our regression analysis, this study employs two distinct methods to mitigate the confounding effects of extraneous factors. The detailed results are outlined in Table 9. Firstly, provinces are considered pivotal conduits for macroeconomic policy enforcement, resource distribution, and localized cultural norms; their inherent disparities can exert both direct and indirect influences on the decision-making processes of microeconomic units. To counteract any potential disruption from regional factors on the interplay between core economic variables, we incorporate three provincial control variables into our analysis: the marketization index (FINDEVE), per capita fixed asset investment (INVP), and supplier concentration (CTION). The resulting regression outputs are presented in columns (1) and (2). Importantly, even after considering these control variables, the regression results align with our primary findings, indicating that regional factors do not significantly alter the conclusions of this investigation. Secondly, we exclude data from the financial crisis period to eliminate its potential interference with the regression outcomes. Specifically, we omit samples from 2007 and 2008, and provide detailed regression results subsequent to this exclusion in columns (3) and (4). Our research conclusions remain consistent with earlier findings, confirming the robustness of our core regression conclusions.

**Table 9:** Eliminate the influence of other factors.

VARIABLES	(1)	(2)	(3)	(4)
	TFP_LP	TFP_FE	TFP_LP	TFP_FE
ROBOT	0.0815***	0.1548***	0.0810***	0.1510***
	(0.0129)	(0.0139)	(0.0111)	(0.0119)
SIZE	0.6448***	0.8141***	0.6540***	0.8324***
	(0.0142)	(0.0152)	(0.0127)	(0.0135)
ROA	1.8596***	1.8530***	1.9492***	1.9646***
	(0.2134)	(0.2205)	(0.2331)	(0.2473)
TOBINQ	−0.1342***	−0.1845***	−0.1366***	−0.1888***
	(0.0076)	(0.0083)	(0.0071)	(0.0077)
AGE	0.0075***	0.0086***	0.0076***	0.0088***
	(0.0017)	(0.0017)	(0.0017)	(0.0017)
CASH	−0.0470	−0.0764	−0.1227**	−0.1817***
	(0.0666)	(0.0696)	(0.0615)	(0.0647)
GPM	−1.2512***	−1.3303***	−1.2556***	−1.3398***
	(0.1104)	(0.1151)	(0.1195)	(0.1274)
MFEE	−6.2925***	−6.5828***	−6.3053***	−6.5568***
	(0.2133)	(0.2222)	(0.2063)	(0.2158)
TANG	−1.1051***	−0.3236***	−1.0777***	−0.2968***
	(0.0785)	(0.0825)	(0.0728)	(0.0760)
FINDEVE	0.0144***	0.0133***		
	(0.0047)	(0.0049)		
INVP	−0.0197***	−0.0172**		
	(0.0075)	(0.0079)		
CTION	−0.0022***	−0.0031***		
	(0.0005)	(0.0005)		
Constant	−4.4491***	−5.8800***	−4.9515***	−6.6451***
	(0.3287)	(0.3504)	(0.2980)	(0.3155)
Observations	12,344	12,344	15,372	15,372
R-squared	0.8241	0.8706	0.8260	0.8720
Industry	YES	YES	YES	YES
Year	YES	YES	YES	YES

#### 4.3.6. IV estimation.

Industrial robot application may be affected by omitted variables or reverse causality, which raises concerns about endogeneity. For instance, enterprise with higher total factor productivity (TFP) often incorporate industrial robots more extensively into their production and operational procedures, potentially leading to a reverse causality issue. To address this concern, we adopt the methodology used by Acemoglu and Restrepo (2020) [3], utilizing the penetration rate of industrial robots in the United States as an instrumental variable and employing a two-stage least squares (2SLS) estimation approach. The rationale for selecting this variable is twofold: Firstly, during the study period, both the United States and China demonstrated comparable levels of progress and application in industrial robots while following similar developmental paths. Secondly, as a global leader in robotics embodying industry-wide technological trends, the United States’ influence on enterprises’ TFP is expected to reflect solely exogenous technological progress and spillover effects. Consequently, the impact of the U.S. industrial robot penetration rate on Chinese enterprises’ TFP primarily mirrors analogous industries’ technological characteristics, meeting the relevance requirement for an instrumental variable. Moreover, it remains largely independent of other domestic factors that influence industrial robot application in China while fulfilling the exogeneity requirement and thus mitigating endogeneity concerns. The empirical results are summarized in Table 10. Specifically, column (1) presents the first-stage estimation results revealing a notably positive regression coefficient between the instrumental variable (USROBOT_IV) and the independent variable (ROBOT), which aligns with the relevance condition. Columns (2) and (3), respectively utilizing Levinsohn-Petrin(LP) method and fixed-effects method, present second-stage TFP estimation results. Both sets of findings indicate significantly positive regression coefficients for industrial robot application consistent with baseline regression results, confirming both robustness of our conclusions as well as alleviation of endogeneity concerns. Furthermore, we continued to use the European robots as the instrumental variable, and the results in Table 11 are consistent with the previous conclusion, confirming the existing conclusion.

**Table 10:** The result of using American robots as IV.

VARIABLES	(1)	(2)	(3)
	First stage	Second stage	Second stage
	ROBOT	TFP_LP	TFP_FE
USROBOT_IV	0.0162***		
	(0.0007)		
ROBOT		0.0368**	0.0697*
		(0.0153)	(0.0381)
SIZE	0.7683***	0.7447***	1.0029***
	(0.0047)	(0.0274)	(0.0296)
ROA	−0.7838***	1.8262***	1.7591***
	(0.1138)	(0.0977)	(0.1057)
TOBINQ	−0.2119***	−0.1614***	−0.2359***
	(0.0039)	(0.0081)	(0.0088)
AGE	0.0056***	0.0082***	0.0100***
	(0.0009)	(0.0007)	(0.0008)
CASH	−0.1107***	−0.1241***	−0.1935***
	(0.0390)	(0.0322)	(0.0348)
GPM	−0.4141***	−1.3019***	−1.4264***
	(0.0495)	(0.0428)	(0.0462)
MFEE	−0.7217***	−6.4743***	−6.8017***
	(0.0830)	(0.0721)	(0.0780)
TANG	2.6768***	−0.7530***	0.3024***
	(0.0349)	(0.0988)	(0.1068)
Cons	−17.1408***	−6.7174***	−10.1635***
	(0.1099)	(0.6125)	(0.6622)
Observations	16,103	16,103	16,103
R-squared	0.8850	0.8226	0.8638
Industry FE	YES	YES	YES
Year FE	YES	YES	YES
Cragg-Donald Wald F	536.93		
Kleibergen-Paap rk Wald F	520.64		

**Table 11:** The result of using European robots as IV.

VARIABLES	(1)	(2)	(3)
	First stage	Second stage	Second stage
	ROBOT	TFP_LP	TFP_FE
EUROBOT_IV	0.0021***		
	(0.0001)		
ROBOT		0.0697*	0.1314***
		(0.0381)	(0.0411)
SIZE	0.7685***	0.8175***	1.0029***
	(0.0048)	(0.0319)	(0.0296)
ROA	−0.7878***	1.7450***	1.7591***
	(0.1142)	(0.1013)	(0.1057)
TOBINQ	−0.2117***	−0.1815***	−0.2359***
	(0.0039)	(0.0093)	(0.0088)
AGE	0.0056***	0.0087***	0.0100***
	(0.0009)	(0.0008)	(0.0008)
CASH	−0.1204***	−0.1351***	−0.1935***
	(0.0392)	(0.0329)	(0.0348)
GPM	−0.4130***	−1.3395***	−1.4264***
	(0.0497)	(0.0444)	(0.0462)
MFEE	−0.7114***	−6.5402***	−6.8017***
	(0.0833)	(0.0750)	(0.0780)
TANG	2.6720***	−0.4992***	0.3024***
	(0.0350)	(0.1139)	(0.1068)
Cons	−17.1373***	−8.3440***	−10.1635***
	(0.1103)	(0.7118)	(0.6622)
Observations	16,103	16,103	16,103
R-squared	0.8841	0.8148	0.8638
Industry FE	YES	YES	YES
Year FE	YES	YES	YES
Cragg-Donald Wald F	409.83		
Kleibergen-Paap rk Wald F	400.45		

## 5. Mechanism tests

To achieve a deeper understanding of the mechanism underlying the impact of industrial robots on enterprises’ TFP, this section employs a mediation effect model. This model is used to explore the various channels through which industrial robots affect TFP, with a specific focus on dimensions such as talent aggregation and innovation capacity. The aim is to clarify the inherent connections within this relationship and provide empirical support for promoting enterprises’ transformation and achieving high-quality development.

### 5.1. Talent aggregation

The primary manifestation of corporate human capital lies in employees who possess high levels of educational achievement, technical expertise, and skill proficiency. For clarity, highly educated employees (HR1) are defined as the ratio of those holding bachelor’s degrees or higher within the overall workforce; highly technical employees (HR2) represent the proportion of technical personnel in the total workforce; and highly skilled employees (HR3) signify the fraction of research and development (R&D) staff within the entire workforce. Table 12 outlines the results of the mechanism test conducted to assess talent aggregation. Specifically, columns (1) to (3) examine the influence of industrial robot application on corporate human capital. The findings reveal that the coefficients estimating the effect of applying industrial robots on human capital show significant positivity, indicating that applying industrial robots significantly enhances the quality of corporate talent. These findings suggest that applying industrial robots has the potential to elevate the level of corporate human capital and subsequently boost an enterprise’s total factor productivity. This evidence supports the “talent aggregation effect” associated with industrial robots and confirms Hypothesis 2 proposed in this research.

**Table 12:** Mechanism test results.

VARIABLES	(1)	(2)	(3)	(4)	(5)
	HR1	HR2	HR3	PAT1	PAT2
ROBOT	0.0035**	0.0041***	0.0069***	0.2215***	0.2561***
	(0.0017)	(0.0013)	(0.0009)	(0.0289)	(0.0279)
SIZE	0.0201***	0.0083***	−0.0048***	0.5846***	0.5981***
	(0.0025)	(0.0019)	(0.0012)	(0.0350)	(0.0345)
ROA	0.1424***	0.0824***	−0.0783***	3.3311***	2.6034***
	(0.0383)	(0.0300)	(0.0278)	(0.4692)	(0.4246)
TOBINQ	0.0007	0.0002	0.0041***	−0.1924***	−0.1477***
	(0.0016)	(0.0011)	(0.0011)	(0.0162)	(0.0152)
AGE	0.0035***	0.0031***	−0.0014***	−0.0109**	−0.0040
	(0.0004)	(0.0003)	(0.0002)	(0.0044)	(0.0045)
CASH	0.0080	−0.0045	−0.0328***	−0.0987	−0.1629
	(0.0150)	(0.0119)	(0.0087)	(0.1487)	(0.1457)
GPM	−0.0858***	−0.0553***	0.0540***	−0.6424***	−0.5323**
	(0.0184)	(0.0145)	(0.0141)	(0.2226)	(0.2076)
MFEE	0.0684*	0.0142	0.2115***	1.1421***	2.0093***
	(0.0373)	(0.0280)	(0.0218)	(0.4020)	(0.3864)
TANG	−0.0770***	−0.0301***	−0.1169***	−1.0104***	−1.1335***
	(0.0143)	(0.0114)	(0.0080)	(0.2043)	(0.1859)
Constant	−0.4737***	−0.2171***	0.1380***	−11.2541***	−12.3907***
	(0.0553)	(0.0415)	(0.0270)	(0.7802)	(0.7651)
Observations	16,103	16,103	16,103	16,103	16,103
R-squared	0.1905	0.1832	0.5245	0.4410	0.4309
Industry	YES	YES	YES	YES	YES
Year	YES	YES	YES	YES	YES

### 5.2. Innovation ability

The evaluation of a enterprises’ innovative capacity often depends on its patent portfolio. In this study, we use the logarithmic transformations of two key metrics to measure its innovative prowess: the overall number of patent applications submitted by the company (PAT1) and the total count of invention patent applications filed (PAT2). The results of the mechanism test related to innovative capacity can be found in columns (4) and (5) of Table 12. Our analysis reveals that the coefficients estimating the effect of industrial robot application on the quantity of corporate patents are significantly positive. This indicates that applying industrial robots notably enhances enterprises’ innovative capacity. These findings imply that industrial robot application acts to bolster an enterprise’s innovative capabilities, ultimately leading to an increase in its TFP. This evidence supports the existence of an “innovation effect” associated with industrial robots and validates the third hypothesis proposed in this research.

## 6. Heterogeneity analysis

To achieve a comprehensive understanding of how the application of industrial robots affects total factor productivity, this section undertakes an exhaustive examination of the various impacts exerted by such application on TFP. The objective is to provide stronger empirical evidence supporting the overall enhancement of TFP.

### 6.1. The heterogeneity of production efficiency

The interplay between production efficiency and the application of industrial robots in relation to TFP is the focus of this section. In scenarios where enterprises already exhibit high levels of production efficiency, the application of industrial robots emerges as a pivotal catalyst for further augmenting their TFP. Firstly, considering technological compatibility and synergistic advantages, enterprises characterized by high production efficiency typically possess well-established production technologies and streamlined process systems (Firth et al., 2015) [36]. By incorporating industrial robots, these enterprises can seamlessly blend and refine their existing production workflows, demonstrating heightened technological assimilation and innovation prowess. Leveraging the precision and efficiency of industrial robots, these enterprises can drastically reduce errors and waste during production, thereby optimizing resource allocation and enhancing TFP. Secondly, enterprises with superior production efficiency often excel in the efficient allocation of labor and capital. The application of industrial robots facilitates the substitution of low-skilled labor, prompting enterprises to expedite the upgrading of their human capital and foster an effective integration of highly skilled labor with advanced capital (Goos et al., 2014) [37]. This integration not only strengthens the enterprise’s innovative capacity but also sustains continuous enhancement in TFP. Consequently, this paper predicts that within high-efficiency enterprises, the application of industrial robots will have a more pronounced effect on TFP.

The methodology for calculating labor productivity (LP) involves subtracting non-operating income from operating income and then dividing the result by the total workforce, with data categorized based on the median. Regression results related to production efficiency are presented in Table 13, where columns (1) and (2) display estimated outcomes of TFP derived using the LP approach and calculated through the fixed-effects method, respectively. The research findings reveal that coefficients of the interaction term between industrial robot application and enterprises’ production efficiency (ROBOT×LP) are significantly positive at a level of 1%, indicating that within enterprises with high production efficiency, the impact of industrial robot application on stimulating TFP is more substantial, aligning with hypotheses posited in this study.

**Table 13:** Heterogeneity analysis results.

VARIABLES	(1)	(2)	(3)	(4)	(5)	(6)
	TFP_LP	TFP_FE	TFP_LP	TFP_FE	TFP_LP	TFP_FE
ROBOT	0.0639***	0.1238***	0.0511***	0.1154***	0.0554***	0.1179***
	(0.0107)	(0.0115)	(0.0108)	(0.0115)	(0.0104)	(0.0111)
LP	0.1139***	0.1012***				
	(0.0201)	(0.0211)				
ROBOT×LP	0.0262***	0.0385***				
	(0.0082)	(0.0087)				
SOE			0.0931***	0.1284***		
			(0.0279)	(0.0291)		
ROBOT×SOE			0.0288***	0.0221**		
			(0.0100)	(0.0105)		
TECH					−0.0814	−0.1318**
					(0.0646)	(0.0669)
ROBOT×TECH					0.0664***	0.0664***
					(0.0120)	(0.0129)
SIZE	0.6722***	0.8558***	0.6498***	0.8328***	0.6485***	0.8333***
	(0.0122)	(0.0130)	(0.0123)	(0.0130)	(0.0128)	(0.0137)
ROA	1.7363***	1.7349***	1.9960***	2.0227***	1.9423***	1.9570***
	(0.2189)	(0.2315)	(0.2271)	(0.2410)	(0.2227)	(0.2364)
TOBINQ	−0.1424***	−0.1964***	−0.1377***	−0.1920***	−0.1335***	−0.1877***
	(0.0069)	(0.0076)	(0.0070)	(0.0076)	(0.0072)	(0.0079)
AGE	0.0072***	0.0083***	0.0054***	0.0063***	0.0074***	0.0086***
	(0.0016)	(0.0017)	(0.0016)	(0.0017)	(0.0017)	(0.0017)
CASH	−0.0939	−0.1468**	−0.1321**	−0.1867***	−0.1197*	−0.1779***
	(0.0595)	(0.0625)	(0.0594)	(0.0622)	(0.0615)	(0.0648)
GPM	−1.2246***	−1.3084***	−1.2264***	−1.3076***	−1.2820***	−1.3686***
	(0.1132)	(0.1202)	(0.1163)	(0.1239)	(0.1146)	(0.1222)
MFEE	−6.3621***	−6.6184***	−6.3581***	−6.6074***	−6.4413***	−6.7023***
	(0.1991)	(0.2077)	(0.2008)	(0.2095)	(0.2041)	(0.2139)
TANG	−0.7663***	0.0426	−1.0494***	−0.2549***	−1.1249***	−0.3254***
	(0.0747)	(0.0780)	(0.0694)	(0.0725)	(0.0743)	(0.0780)
Constant	−5.2319***	−7.0116***	−4.6450***	−6.4316***	−4.5231***	−6.2789***
	(0.2839)	(0.3004)	(0.2806)	(0.2961)	(0.3035)	(0.3237)
Observations	16,103	16,103	16,075	16,075	16,103	16,103
R-squared	0.8308	0.8750	0.8296	0.8742	0.8269	0.8717
Industry	YES	YES	YES	YES	YES	YES
Year	YES	YES	YES	YES	YES	YES

### 6.2. The heterogeneity of nature of property rights

The nature of property rights within enterprises has an influence on the impact of industrial robot application on total factor productivity. For example, state-owned enterprises (SOEs) generally have substantial capital strength and resource bases, providing a solid foundation for applying industrial robots. This extensive resource support ensures that SOEs can fully utilize the technological capabilities of industrial robots during deployment, thereby exerting a significant positive effect on TFP. On the other hand, SOEs often bear the heavy responsibility of national economic strategies and industrial advancement, making them more inclined to embrace cutting-edge technologies to drive shifts in production methods. Due to excessive employee recruitment, SOEs face higher labor costs and limited funds for innovation, making the application of industrial robots even more crucial for enhancing production efficiency and improving human capital levels. The application of industrial robots aligns with the inherent needs of SOEs for technological innovation and industrial upgrading, reducing resource waste and labor expenses, ultimately leading to an increase in TFP. Therefore, this study predicts that the contribution of applying industrial robots to TFP will be more prominent within SOEs.

The categorization of property rights as SOE is designated as 1, and 0 otherwise. Specifically, columns (3) and (4) in Table 13 delineate the regression outcomes after considering the nature of property rights. Column (3) presents the evaluation results of TFP computed using the LP method, while column (4) reveals the assessment results of TFP determined through the fixed effects method. The research findings indicate that both coefficients of the interaction term between industrial robot application and the nature of property rights (ROBOT×SOE) are positive. This implies that within SOEs, the application of industrial robots has a more significant impact on enhancing TFP, aligning with the expectations outlined in this study.

### 6.3. The heterogeneity of technical ability

The technological prowess of enterprises plays a crucial role in shaping the impact of industrial robot application on TFP. Firstly, the industrial robots have high technological complexity (Zhang et al., 2022) [38], high-tech enterprises positioned at the forefront of technological advancement exhibit formidable capacities for assimilating and transforming novel technologies. They can seamlessly integrate industrial robots into their production workflows, thereby maximizing the use of their technological edge. Secondly, high-tech enterprises often demonstrate high levels of capital and technology intensity, which require significant investment in human capital. While industrial robots cannot easily replace such human capital (Goos et al., 2014) [37], their strong capabilities in independent innovation and research and development investment can continuously drive the extensive application and technological advancement of industrial robots within production processes, thereby enhancing the productivity and innovative potential of highly skilled workers. Therefore, this study hypothesizes that the impact of industrial robot application on TFP is more pronounced in high-tech enterprises.

High-tech enterprises (TECH) are coded as 1, while non-high-tech enterprises are coded as 0. Table 13 presents the regression outcomes after considering technological capability, specifically in columns (5) and (6). Column (5) displays the estimated results of TFP derived using the LP method, whereas column (6) presents the estimated results of TFP computed using the fixed-effects method. The research findings indicate that both coefficients of the interaction term between industrial robot application and technological capability (ROBOT×TECH) are positive. This indicates that within high-tech enterprises, the deployment of industrial robots has a more significant impact on enhancing TFP, which aligns with the expectations stated in this study.

## 7. Conclusion and policy implications

### 7.1. Conclusion

The emergence of the Fourth Industrial Revolution, driven by advancements in information technology, emphasizes the crucial role of digital technology as a vital tool for facilitating global industrial transformation and upgrading. It is essential for businesses to transition from their current production paradigms by embracing industrial robots to achieve high-quality growth. This study uses micro-panel data from Chinese A-share listed manufacturing enterprises spanning the period from 2007 to 2022 to comprehensively analyze the interaction and underlying mechanisms between the application of industrial robots and total factor productivity. Our findings suggest that the application of industrial robots has a positive influence on firm-level TFP, demonstrating a sustained effect that remains robust across various tests of robustness. Upon delving into the mechanisms, we find that the application of industrial robots augments TFP by elevating levels of human capital and nurturing innovation capabilities, thereby affirming its “talent aggregation effect” and “innovation effect”. Furthermore, heterogeneity analysis reveals that the stimulatory effect of applying industrial robots on TFP is more pronounced among high labor productivity enterprises, state-owned enterprises, and high-tech enterprises. This research elucidates the pathway through which industrial robots impact enterprises’ TFP, enriching our understanding of the economic implications of applying industrial robots and carrying significant practical significance for promoting intelligent transformation in manufacturing.

### 7.2. Policy implications

Firstly, industrial robots, as a pivotal element in contemporary advanced manufacturing technology, have gained increasing prominence in driving the transformation and upgrading of manufacturing. Their widespread deployment not only embodies technological advancements in manufacturing but also acts as a primary catalyst for shifting from low-end processing to high-end intelligent manufacturing. Consequently, government departments should adopt a macro-level perspective, undertake comprehensive top-level design for the industrial robot sector, and vigorously advance the development and implementation of related technologies. At the policy level, it is crucial to devise rational fiscal and taxation policies that offer financial subsidies and tax reliefs for technological research, development, adoption, and application of industrial robots. Concurrently, establishing relevant incentive mechanisms to enhance motivation for core technical talents is essential. Different types of enterprises should be guided to fully harness industrial robots for industrial upgrading purposes. Tailored technical assistance should be provided taking into account the distinct characteristics and needs of various industries thereby guiding manufacturing enterprises to expedite the intelligent transformation of their production lines and achieve high-quality economic growth.

Secondly, with the deep integration of digital technology and industrial robots, artificial intelligence technology is expected to further expand its applications across various industries, exerting a significant impact. This trend will undoubtedly affect job markets and pose challenges for employment landscapes. While applying industrial robots can enhance enterprises’ human capital levels, it may also reduce competitiveness among low-skilled workers. To tackle this issue, the government should take a leading role or provide support in establishing talent development platforms focused on industrial robotics. These platforms would offer cutting-edge research facilities and work environments. By implementing talent attraction programs and offering competitive salaries, as well as providing research funding support, the government should encourage overseas talents to return or relocate to China for engaging in research and application activities related to industrial robots. To assist low-skilled talents, the government should increase investment in vocational education, intensify training for low-skilled workers, enhance their labor skills and professional competencies, and strengthen their competitiveness in the labor market.

Lastly, as traditional growth drivers progressively decline, enhancing TFP has emerged as a crucial avenue for manufacturing enterprises to achieve high-quality development. Enterprises should increase investments in technologies such as industrial robots and artificial intelligence, fully exploit their capabilities, elevate workforce productivity, and expedite the integration of internal knowledge and technology. This will create conducive conditions for propelling transformation and upgrading while improving economic returns. Simultaneously, enterprises should also reinforce employee training and potential development to enhance the quality of human capital by improving employee skills and attributes further stimulating the increase in TFP. Moreover, enterprises should revamp their technological innovation models, strengthen collaboration with universities and research institutions, and leverage the collective power of collaborative innovation to augment TFP and drive high-quality economic growth.

### 7.3. Discussion

The global manufacturing industry is currently undergoing a pivotal phase of intelligent transformation, where decisions regarding industrial robots not only exert significant influence on individual enterprises but also impact the entire industry. A comprehensive exploration of these diverse impacts from different perspectives can offer policymakers valuable empirical evidence. This study empirically evaluates the effects of industrial robots on enterprises’ TFP and its underlying mechanisms from the viewpoint of micro-enterprises by using panel data obtained from Chinese manufacturing enterprises. Additionally, it conducts heterogeneity analyses based on enterprise characteristics. This research carries substantial theoretical and practical implications for China and other emerging market economies when formulating strategies for robot development.

Firstly, the baseline regression results indicate that the application of industrial robots has a positive impact on enterprises’ TFP. Furthermore, this effect is not only significant in the short term but also remains strong over the following one to two years, suggesting that applying industrial robots can bring immediate economic benefits for enterprises and lay a solid foundation for their future development. The findings of this study are generally consistent with previous research on the influence of industrial robots on TFP in developed economies (Acemoglu and Restrepo, 2020; Koch et al., 2021) [3,9] and limited research on the impact of industrial robot application on productivity in China (Aghion et al., 2015) [29]. As a result, this paper not only expands the research scope of industrial robots and TFP but also provides empirical evidence from China to address the “Solow paradox” associated with industrial robots. Additionally, this study offers practical insights for China and other emerging economies in formulating strategies for industrial robot development and implementing intelligent transformations in manufacturing.

Secondly, the mechanism analysis reveals that the influence of industrial robots on TFP is transmitted through two channels: enhancing levels of human capital and fostering innovation capabilities. This supports the “talent aggregation effect” and the “innovation effect” of industrial robots. The study confirms the effective role of industrial robots in improving enterprises’ human capital structure. High-level human capital facilitates the enhancement of knowledge density networks within enterprises and promotes knowledge spillover effects, thereby assisting in establishing internal innovation networks within groups and strengthening enterprises’ innovation capabilities. This has significant practical value in addressing the lack of technological innovation in manufacturing enterprises and exploring opportunities for transformation and development.

Lastly, the analysis of heterogeneity across various types of enterprises indicates that the application of industrial robots has a more significant impact on TFP in enterprises characterized by high production efficiency, state ownership, and advanced technology. This finding confirms the differentiated influence of industrial robot application on different enterprises, offering practical guidance to governmental agencies and businesses in developing customized strategies for “machine substitution” and systematically fostering the growth of the robotics industry.

However, although this research provides valuable insights into the relationship between industrial robots and TFP, it has certain limitations. Firstly, due to its focus on manufacturing as a primary sector for robot application, this study only examines how industrial robots affect TFP in China without investigating their impact on other emerging economics. In future research, we intend to broaden our investigation by exploring how industrial robots influence various industries. Secondly, concerning mechanisms examined in this study are limited to talent aggregation and innovation capabilities; future studies should consider exploring additional factors that contribute to understanding the intricate interaction between robot application and TFP from different perspectives.

## Supporting information

S1 File The supporting information is the full text of the paper, including chart information, providing comprehensive materials and data. (ZIP)
